# Synovial Lipomatosis of the Glenohumeral Joint

**DOI:** 10.1155/2016/4170923

**Published:** 2016-08-01

**Authors:** Shaul Beyth, Ori Safran

**Affiliations:** Orthopedic Surgery Department, Hadassah Medical Center, 91120 Jerusalem, Israel

## Abstract

Synovial lipomatosis (also known as lipoma arborescens) is a rare and benign lesion affecting synovium-lined cavities. It is characterized by hyperplasia of mature fat tissue in the subsynovial layer. Although the most commonly affected site is the knee joint, rarely additional locations such as tendon sheath and other joints are involved. We present a case of synovial lipomatosis of the glenohumeral joint in a 44-year-old man. The clinical data radiological studies and histopathologic results are described, as well as a review of the current literature.

## 1. Introduction

Synovial lipomatosis is a rare and benign lesion affecting synovium-lined cavities [1]. It most commonly affects the knee, but in rare cases also the hip, elbow, wrist, ankle, tendon sheath, and shoulder. This intra-articular condition of unknown etiology is marked by villous synovial proliferation with replacement of the subsynovial tissue by adipose tissue and mature fat cells [2]. Synovial lipomatosis may present itself as an inflammatory condition of the joint with or without systemic manifestations. Several cases with shoulder involvement were reported; only three of them related to the glenohumeral joint while the others involved the subacromial space. In two of these cases bone involvement was reported [2, 3]. We present a rare case of late posttraumatic synovial lipomatosis of the glenohumeral joint in a 44-year-old chef. Diagnosis was established on the basis of clinical data, imaging studies, and histopathology. A pertinent literature review is provided.

## 2. Case Presentation

A 44-year-old right hand dominant chef referred to the clinic complaining of recurrent episodes of right shoulder pain with prolonged asymptomatic periods between them. He described an episode that occurred four years earlier in which he was hospitalized for suspected glenohumeral joint infection. The suspected diagnosis was then based on a combination of severe shoulder pain, globally limited range of motion, fever of 38°C, and elevated ESR and CRP values (50 mm/hour and 12 mg%, resp.). At that time an ultrasound examination revealed a large amount of fluid in the glenohumeral joint and the bicipital tendon sheath. A synovial fluid sample was aspirated and a Gram stain showed only a few white blood cells but was negative for the presence of bacteria. The cultures were also negative. The patient's condition improved under nonsteroidal anti-inflammatory treatment and he was discharged. Three years later he had a similar episode for which he was treated as before.

He came to our outpatient clinic a few weeks later. His physical examination at that point was unremarkable. There was normal active range of motion as well as normal cuff strength and no signs of instability or impingement. Tests for subacromial and bicep irritation were negative.

**Figure 1:**
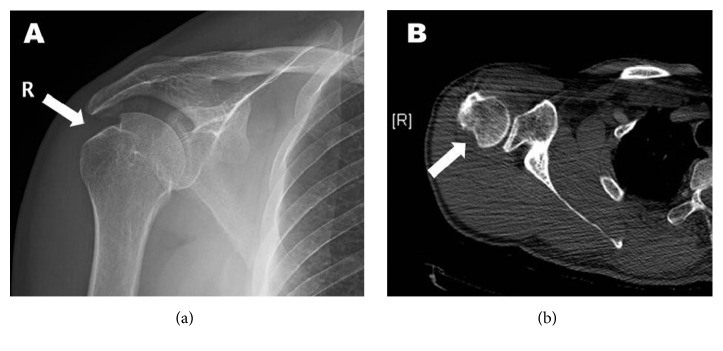
Preoperative imaging of the right shoulder. A medium size Hill-Sachs lesion is demonstrated in the posterosuperior aspect of the humeral head (arrow) in a plain AP radiograph of the shoulder (a) and in an axial plain image of a CT scan (b), suggesting previous anterior dislocation of the glenohumeral joint.

Plain radiographs as well as a CT scan which were performed a few years earlier demonstrated a medium size Hill-Sachs lesion, without blunting of the anterior inferior glenoid (Figure 1). Only when confronted with the findings did the patient recall sustaining an indirect trauma to the shoulder some 20 years earlier for which he was unable to provide any documentation.

**Figure 2:**
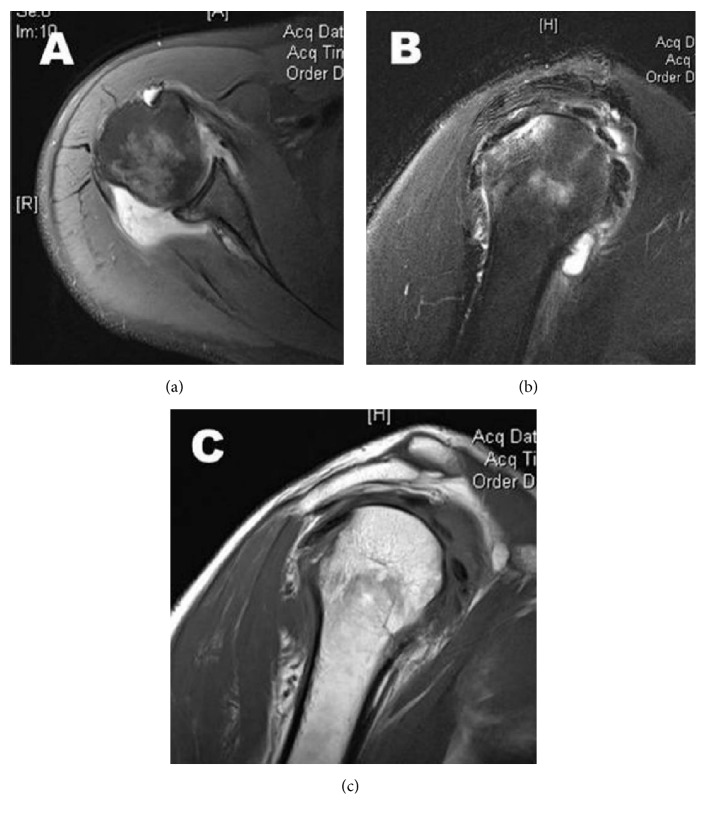
MRI of the right shoulder. (a) Axial proton density fat suppressed weighted image showing regions of low signal intensity within the joint effusion. (b) Sagittal proton density fat suppressed weighted image showing suppression of the signal intensity of the lesions, similar to subcutaneous fat. Bone erosion is present at the superior aspect of humeral head representing a Hill-Sachs fracture. (c) Sagittal T1-weighted image showing a large joint effusion and heterogeneous signal intensity within the periphery of the joint, suggestive of synovial hyperplasia.

An MRI examination revealed large joint effusion and heterogeneous signal intensity within the periphery of the joint, suggestive of synovial hyperplasia (Figure 2).

Due to the persistence of the symptoms the patient underwent arthroscopy of his right shoulder which demonstrated abundant yellowish villous synovial tissue in the glenohumeral region. The rotator cuff tendons as well as the articular cartilage were intact. The subacromial space was not involved. After samples were obtained, arthroscopic synovectomy was performed.

**Figure 3:**
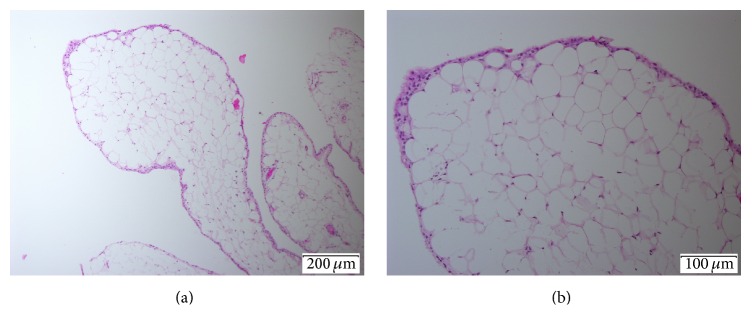
Histopathological photomicrographs. Hematoxylin and eosin stained sections of samples obtained from the glenohumeral joint during arthroscopy showing synovial lined villous proliferation which is diffusely infiltrated by mature fat cells while the periphery is lined by hypertrophic synovial cells and infiltrated by chronic inflammatory cells (bar size at lower right corner of images indicates 200 *μ*m panel (a) and 100 *μ*m panel (b)).

Histopathological examination of the retrieved tissue showed fragments of synovial villi with no acute inflammation or infection, with evidence of lipomatous metaplasia (Figure 3).

The patient's postoperative course was uneventful. Twelve months after surgery his pain has diminished with no recurrence of inflammatory episodes to date.

## 3. Discussion

Synovial lipomatosis is a rare benign lesion affecting synovium-lined cavities. It most commonly affects the knee [2] but rarely affects the hip [4, 5], elbow [6, 7], wrist [8], ankle [1, 9], tendon sheath [2, 6], and shoulder [3, 10, 11]. Although synovial lipomatosis is most commonly a monoarticular condition, several cases of multifocal disease have been reported [12, 13].

Since it was first described by Hoffa [14] in 1904, only several cases of synovial lipomatosis of the shoulder were reported, of which only three were related to the glenohumeral joint [3, 10, 11] and the others were describing subacromial space lesions [15–17]. Interestingly, although synovial lipomatosis is primarily a soft tissue condition, in two of these cases, bone erosion in young healthy patients was also present [3, 11]. The third reported patient was a 90-year-old woman, and therefore joint erosion may be assumed, although it was not reported [3].

In 1995 Laorr et al. published the first description of synovial lipomatosis of the shoulder in a 90-year-old patient. The diagnosis was based on clinical findings and imaging, and the patient was treated nonoperatively [3]. In et al. reported in 2008 a case of glenohumeral synovial lipomatosis diagnosed by arthroscopy in a 22-year-old male patient. The authors noticed bone erosion and arthritic changes of the joint and assumed a causative relation by which the synovial metaplasia induces degenerative joint disease [11]. One year later Chae et al. reported a similar case involving a 36-year-old male patient in whom the affected shoulder was also deranged [10]. In their report they emphasize the seven-year delay in diagnosis that may be attributed to the insidious nature of the disease.

Synovial lipomatosis may equally affect males and females, between the second and the ninth decades of life. It has been suggested that adolescent patients present an idiopathic primary form of the disease, whereas older individuals, young and older adults, exhibit a secondary process. Less than a handful of multifocal cases were described in young patients. The etiology was suggested to be linked to systemic conditions in some reports, specifically to fat metabolism due to coexisting short bowel syndrome in one case [18] and to chronic inflammatory diseases such as rheumatoid arthritis in another [12]. This may follow traumatic or degenerative injury to the joint with resultant chronic irritation of the synovium [9, 19]. This is somewhat contrary to the theory raised by In et al. and Chae et al., who considered the synovial lipomatosis to be the primary lesion and the bony involvement only secondary. Synovial lipomatosis as a reactive process has been further supported by observations made by Ikushima et al. who noticed decreased osteogenic activity and increased adipogenic activity of marrow cells in this condition [20]. The authors suggested that synovial chondromatosis is yet another form of the synovial reaction and supported their theory by the coincidence of the two conditions in two cases, as well as the similar age distribution of the two conditions. They concluded that the spectrum of late synovial reaction to injury may include several forms. In our case we found clear evidence of prior anterior shoulder dislocation that took place well before the onset of symptoms, consistent with this paradigm.

Clinically, synovial lipomatosis usually presents an inflammatory condition of the joint. Manifestations include painless swelling of the involved joint due to accumulation of synovial fluid. In addition, this condition of unknown etiology is marked by villous proliferation consisting of replacement of the subsynovial tissue by adipose tissue with mature fat cells [2]. Lack of pain and systemic manifestations in most cases may explain the long period that usually elapses until diagnosis. Indeed, our case is unique due to the documented significant pain accompanied by fever and systemic inflammatory response. Unfortunately, at that time clinicians were misled by the acute presentation and assumed joint infection, whereas accurate diagnosis was made only at a later stage.

Diagnosis of synovial lipomatosis is confirmed by histology. Although plain radiography and CT scan may not be very helpful, magnetic resonance imaging criteria have been published as an aid to diagnosis. These may include high signal intensity villous or nodular foci on both T1- and T2-weighted images that are suppressed on short tau inversion recovery (STIR) or fat saturation sequences, while the remaining nonfatty component of the hypertrophied synovium displays heterogeneous high signal intensity on T2 or STIR sequences and intermediate-to-low signal intensity on T1-weighted sequences. Degenerative or posttraumatic changes may be seen in the secondary (but not the primary) type of synovial lipomatosis. The subsynovial fatty proliferation usually presents in the diffuse villous form or in the focal nodular frond-like form. A mixed form of the two patterns may also be seen [3, 6, 10, 19, 21].

In 2003 Vilanova et al. reported a series of 33 cases of synovial lipomatosis in 32 knees and one shoulder in which the lesion was located in the subacromial bursa. Only 12 lesions were confirmed by histology, diagnosis of the remaining 21 joints based on MRI findings only [19]. These findings included joint effusion in all cases and other unspecified degenerative findings in all but two knees, whereas other intra-articular lesions were less common. A cuff tear identified by MRI was assumed to be related to the synovial lipomatosis of the shoulder case, as suggested before [15, 17].

Differential diagnosis of synovial lipomatosis of the shoulder should include pigmented villonodular synovitis, rheumatoid arthritis, tuberculous arthritis gouty arthropathy and synovial osteochondromatosis. Although clinically these may be indistinguishable from synovial lipomatosis, correct diagnosis can be made based on detailed history, laboratory tests of blood, and synovial fluid as well as imaging, particularly by magnetic resonance.

Asymptomatic and therefore undiagnosed cases of synovial lipomatosis should be assumed to exist. When symptomatic, synovial lipomatosis is commonly treated nonoperatively by physical therapy and anti-inflammatory medications aimed at controlling the reactive episode. Failure of these modalities may indicate the need for invasive measures.

Treatment of synovial lipomatosis by radionuclide therapy in one case yielded significant reduction of lesion volume at six months [22]. No clinical benefit was confirmed. An additional case treated by radionuclide therapy was described by Erselcan et al. Here a 36-year-old woman was treated with intra-articular administration of yttrium-90 in conjunction with 40 mg methylprednisolone acetate. The authors described the clinical benefit at one-year follow-up, supported by MRI findings [23]. Although radionuclide therapy was reported for these two cases, the treatment of choice for synovial lipomatosis is arthroscopic radical synovectomy. This minimally invasive procedure facilitates early postoperative recovery. Recurrence of synovial lipomatosis after synovectomy is uncommon.

## 4. Conclusions

Synovial lipomatosis (lipoma arborescens) is an uncommon intra-articular condition of unknown etiology, with less than a handful of cases reported in the glenohumeral joint. The etiology is unclear, but synovial lipomatosis was suggested to represent a late reaction of the synovium to injury in young and older adults. Although it commonly presents insidiously with local soft tissue swelling and joint effusion, on rare occasions it may manifest with an acute local and systemic inflammatory response that is difficult to differentiate from septic shoulder. Clinical and radiological evaluation may facilitate diagnosis. Arthroscopic synovectomy may result in prolonged remission of symptoms.
